# Targeting refractory diffuse large B cell lymphoma by CAR-WEE1 T-cells: In vitro evaluation

**DOI:** 10.1007/s00277-024-06134-8

**Published:** 2025-01-17

**Authors:** Hadeer Mohamed Ahmed, Said Salama Moselhy, Magda I. Mohamad, Ahmed F. Soliman, Marwa N. M. Hassan, Nashwa El-Khazragy

**Affiliations:** 1 Department of Biochemistry, Faculty of Science, Ain Shams University, Cairo, 11566 Egypt; 2 Department of Medical Biochemistry and Molecular Biology, Faculty of Medicine, Ain Shams University, Cairo, 11566 Egypt; 3 Department of Clinical Pathology-Hematology and AinShams Medical Research Institute (MASRI), Faculty of Medicine, Ain Shams University, Cairo, 11566 Egypt

**Keywords:** CAR T cells, Diffuse large B-cell lymphoma, Rituximab resistance

## Abstract

Refractory Diffuse Large B-cell Lymphoma (DLBCL) presents a major therapeutic challenge due to its resistance to standard treatments. Engineered T-cells, especially Chimeric Antigen Receptor (CAR) T-cells, have shown promise in overcoming drug resistance. This study investigates the effectiveness of WEE1-engineered T-cells in targeting and eliminating refractory DLBCL in vitro. CAR T-cells were created by transducing a 5th-generation CAR construct designed to recognize WEE1, a surface antigen commonly found on refractory DLBCL cells. The cytotoxic effect of engineered T-cells was tested against Rituximab-resistant DLBCL cells (RR-NU-DUL-1). Apoptosis and cell cycle were evaluated using flow cytometry. Quantitative Real-time PCR (RT-PCR) was used to measure the expression of WEE1, BCL2, and CDK2. The results showed a significant increase in target cell lysis, apoptosis, and necrosis, a significant reduction in the percentage of cells in the G2M phase of the cell cycle, as well as a decrease in gene expression level, indicating strong anti-tumor activity. These findings suggest that CAR T-cell therapy holds great promise for treating refractory DLBCL, offering a potential path for clinical application. This in vitro evaluation highlights the potential of WEE1-engineered T-cells as a targeted treatment strategy for refractory DLBCL, emphasizing their clinical applicability and ability to overcome resistance mechanisms in this aggressive lymphoma subtype.

**Supplementary Information:**

The online version contains supplementary material available at 10.1007/s00277-024-06134-8.

## Introduction

Rituximab resistance in Diffuse Large B-cell Lymphoma (DLBCL) presents a significant challenge, undermining the effectiveness of one of the primary treatments for this aggressive cancer [1, 2]. These resistance mechanisms diminish the therapeutic impact of Rituximab, necessitating the exploration of alternative strategies [3, 4]. One promising solution is CAR T cell therapy engineering to target and destroy cancer cells [5, 6]. CAR T cells can recognize and attack cancer cells with high specificity, potentially overcoming the limitations of Rituximab [7]. The adaptability of CAR T cells to target different antigens offers a versatile approach to combat resistance and improve outcomes for DLBCL patients [8].

CAR T cell therapy, an innovative immunotherapy for lymphoma, has evolved through several generations, each improving upon the previous one [9]. The first-generation CAR T cells were limited by their short lifespan and limited efficacy, possessing a single signaling domain (CD3ζ) [10]. The second generation introduced an additional co-stimulatory domain (CD28 or 4-1BB), significantly enhancing T cell proliferation, persistence, and antitumor activity [5]. The third generation further integrated multiple co-stimulatory domains, increasing potency and durability [11]. The fourth generation, also known as "TRUCKs" (T cells Redirected for Universal Cytokine Killing), incorporates cytokine release to boost immune response in the tumor microenvironment [12]. Most recently, the fifth generation CAR also has a synergistic activation of triple signals from CD3ζ, co-stimulatory molecules, and cytokine-inducing JAK-STAT3/5 pathway [11].

The advantages of CAR T cell therapy are profound, offering high specificity to cancer cells, long-term remission for some patients, and overcoming traditional chemotherapy resistance [13]. However, challenges persist [14, 15], including neurotoxicity and cytokine release syndrome (CRS) [16]. The high cost and complex manufacturing process also limit accessibility [16–18].

In lymphoma, CAR T cell therapy has shown remarkable success, particularly in refractory cases where traditional treatments have failed [19]. It is FDA-approved for certain types of lymphoma, including DLBCL [20, 21]. CAR T-cell therapies targeting specific antigens, such as CD19, have been approved for use in relapsed or refractory diffuse large B-cell lymphoma (DLBCL) patients who have failed two or more lines of systemic therapy. FDA-approved products, including axicabtagene ciloleucel (Yescarta) and tisagenlecleucel (Kymriah), target CD19 on malignant B cells [22, 23]. Ongoing research aims to broaden its application, improve safety profiles, and enhance its efficacy, making CAR T cell therapy a promising approach in the fight against lymphoma [24].

The WEE1 gene encodes a tyrosine kinase that regulates the G2/M checkpoint for the cell cycle [25], ensuring cells do not enter mitosis prematurely [26–28]. In DLBCL, WEE1 is often overexpressed, contributing to the disease's pathogenesis [29]. This overexpression allows cancer cells to bypass normal cell cycle arrest mechanisms, promoting unchecked proliferation. WEE1 inhibits CDK1 activity, leading to cell cycle arrest and preventing apoptosis, thus supporting tumor growth and survival [29, 30]. In the context of Rituximab resistance, WEE1's role in maintaining the DNA damage response and promoting cell survival under stress conditions is significant [31]. This kinase helps lymphoma cells endure the cytotoxic effects of Rituximab, diminishing its therapeutic efficacy [32].

In the current study, we have designed a 5th-generation WEE1-specific CAR T cell approach to target WEE1 epitopes in refractory DLBCL. Regarding efficacy and safety, the CAR T was designed to overcome the immunosuppressive factors and tumor escape phenomena, balance benefits with toxicity, and achieve the highest specificity to tumor cells. In addition, we confirm and validate the molecular signaling pathway of the WEE1 gene in the refractoriness of DLBCL.

The connection between rituximab and the investigated CAR product is based on findings that B cells, particularly rituximab-resistant B cells in DLBCL, often exhibit overexpression of specific proteins. A study by Mathilde et al. using a guilt-by-association analysis of CD20-associated gene probes identified WEE1 as a relevant target in this context. Our hypothesis is that by designing CAR T cells to target B cells expressing the WEE1 protein, we could exploit vulnerabilities in these resistant cells, providing a complementary strategy to overcome rituximab-associated therapeutic failures.

## Materials & methods

### Computational construction of 5th-generation CAR T cells targeting the WEE1 protein

Numerous databases and bioinformatics procedures are employed. First, the ***UniProt protein database*** is used to retrieve the WEE1 protein sequence. The ***Immune Epitope Database (IEDB)*** program is used for epitope mapping [33], which locates possible WEE1 epitopes that CAR T cells can target. The immunogenicity of these epitopes is subsequently assessed using the ***NetMHCpan*** and ***NetCTL*** algorithms, which also predict the epitopes' propensity for binding to MHC molecules [34, 35]. Subsequently, the appropriate single-chain variable fragment (scFv) is developed by searching antibody databases for antibodies that bind to the chosen WEE1 epitopes [36], including the ***Protein Data Bank*** and the ***Structural Antibody Database (SAbDab)*** [37].

Intracellular signaling domains "CD28, 4-1BB, CD3ζ" and an extra IL-2 receptor domain for the fifth-generation construct are included in the CAR build design [38]. The sequences for these domains were designed using the ***NCBI GenBank genome database***. The CAR construct is constructed using computational methods called "***SnapGene***," which guarantees the best codon usage for human cells. ***PyMOL software*** is used for molecular modeling and structural investigation of the CAR construct to guarantee correct folding and functionality [39–41].

The intended CAR construct is then produced and cloned into the vector backbone of pcDNA3.1. To guarantee sequence accuracy and the lack of off-target effects, the ***BLAST*** computational techniques are employed. Finally, the complete CAR sequence is verified and optimized for expression in human T cells using the ***Codon Optimization On-Line (COOL)*** in silico tool [42].

After that, this computer-generated CAR is prepared for synthesis and T-cell transduction to target WEE1 in refractory DLBCL. The pcDNA3.1-WEE1 CAR vector and the sequence of the primer to amplify the WEE1 CAR DNA were provided in **supplementary Table 1**.

### Cell lines and culture conditions

The Rituximab-sensitive human-activated B-cell-like Diffuse Large B-Cell Lymphoma (DLBCL) cell line (NU-DUL-1), obtained from the American Type Culture Collection (***ATCC, Manassas, VA, USA***), was used for this study. Cells were maintained in Roswell Park Memorial Institute Medium 1640 (RPMI-1640) supplemented with 10% fetal bovine serum and penicillin–streptomycin (100 U/mL) at 37 °C with 5% CO2 in a humidified incubator. Human serum, serving as a complement source, was purchased from ***Sigma-Aldrich (St. Louis, MO, USA).*** The culture medium was replaced every three days, and the cells were regularly monitored for morphology and subculture as needed. Subculturing occurred when the cells reached 80% confluency, transferring them to fresh media. Cells from the third passage were used for the experiments.

### Induction of a rituximab-resistant cell line

To induce a Rituximab-resistant cell line, NU-DUL-1 cells at the third passage were maintained in RPMI-1640 medium supplemented with 10% fetal bovine serum and penicillin–streptomycin (100 U/mL) at 37 °C and 5% CO2 in a humidified incubator. The half-maximal inhibitory concentration (IC_50_) of Rituximab for these cells was determined by ***Czuczman MS et al., 2008*** [43]. The cells were gradually exposed to escalating doses of Rituximab (0.1—128 µL/mL) from (***Roche, Basel, Switzerland***)*,* along with human serum from ***(Sigma-Aldrich, St. Luis, MO, USA)***, for 24 h at 37 °C and 5% CO2 in a humidified incubator. Following each 24-h incubation with a specific dose of Rituximab and human serum, based on the protocol by ***Van Meerten et al., 2006*** [44], the cells were centrifuged and retreated with increasing doses of Rituximab. This process continued until a resistant cell line developed. The cells were kept in culture with the drug for several weeks, with the medium and drug being changed as needed. Cell viability was regularly monitored using viability assays to ensure most cells were killed, allowing for the selection of resistant populations.

Once resistant cell populations emerged, a limiting dilution was used to generate single-cell clones. Individual cells were plated into separate wells of a 96-well plate to facilitate the expansion of resistant clones. The characterization and validation of the resistant cell lines involved verifying resistance by comparing the IC_50_ values of the resistant clones to those of the parental cell line. Finally, once the rituximab-resistant cell lines were validated and characterized, they were cryopreserved in multiple vials to ensure their long-term availability.

### Isolating and Activating CD3/CD28 Lymphocytes from Peripheral Blood Mononuclear Cells (PBMCs)

Venipuncture was performed to collect 5 mL venous blood samples from adult volunteers aged 30–35 years. Blood samples were collected in K2EDTA vacutainers. Lymphocytes were isolated from these samples using Ficoll-Hypaque density gradient centrifugation (***Sigma-Aldrich, St. Louis, MO, USA)*** [45]. Activated T cells were then isolated from PBMCs using Dynabeads™ Human T-Activator CD3/CD28 cell-based assays (***Gibco, Life Technologies***). Initially, the Dynabeads were washed twice in phosphate-buffered saline (PBS) containing 0.1% bovine serum albumin (BSA) and 2 mM EDTA at pH 7.4. The washed Dynabeads were resuspended in 10 µL of Optimizer T cell Expansion serum-free medium (SFM) ***(Gibco, Life Technologies)*** and incubated with the cells at 37 °C in a CO2 incubator for 72 h for activation. The culture was monitored daily for cell growth using an automated cell counter. After activation, the Dynabeads were carefully removed using a magnetic stand, and the supernatant, containing the CD3/CD28-activated cells, was gently transferred to a new cell culture flask for downstream applications [46].

### Expansion and culture of CD3/CD28-Activated T-Cells

In a humidified CO2 incubator at 37 °C, 1 × 10^6 CD3/CD28-activated T-cells were seeded in a 24-well culture plate and incubated for 72 h. The cells were maintained in 25 μL Dynabeads Human T-Activator CD3/CD28, 2 µL (30 U/mL) of rIL-2, and 96 μL of Optimizer T cell Expansion serum-free medium (SFM). Daily examinations of cell size and shape were conducted. Signs of cell exhaustion, such as shrinking and reduced proliferation, were monitored. Cells were counted at least twice weekly after thorough resuspension. When cell density exceeded 2.5 × 10^6 cells/mL or the medium turned yellow, cultures were split back to 0.5–1 × 10^6 cells/mL in a culture medium containing 30 U/mL rIL-2 [47].

### Cloning & transduction of activated T cells with CAR gene construct

A DNA construct was used to transduce activated T cells. The pcDNA3.1 + 5.4 kb plasmid vector "*pcDNA 3.1* cloning vector" (cat no: V790-20, ***Invitrogen, ThermoScientific, USA***) was employed to deliver the target CAR gene into mammalian cells via in vitro transfection. The PCR product and *pcDNA3.1(* +*)* plasmid were digested with *EcoRI* and *XbaI* (***Fermentas, Germany***) using 10X Fast Digest buffer (cat no: B64, ***ThermoScientific, USA***). The 60 μL reaction mixture consisted of 20 μL of *pcDNA3.1(* +*)* (25 ng/µL), 5 μL digestion buffer, 2 μL *EcoRI* (10 U/mL), 2 μL *XbaI* (10 U/mL), and 31 μL DNase-free water. The thermal protocol included a two-hour incubation at room temperature followed by heating at 70 °C for 10 min to inactivate the enzymes. The digested product was validated using 0.5% agarose gel electrophoresis [48].

To insert the CAR gene into the *pcDNA3.1*( +) vector, the CAR DNA sequence was ligated to the sticky-ended *pcDNA3.1*( +) using T4 DNA ligase (cat no: EL0011, ***ThermoScientific, USA***). A 20 μL ligation reaction mixture was prepared, containing 1 μL DNA insert (25 ng/μL), 3 μL linearized vector *pcDNA3.1*( +) (50 ng), 2.5 μL T4 DNA ligase (5 U/μL), 2.5 μL T4 DNA ligase buffer, and 11 μL nuclease-free water. The reagents were mixed, centrifuged briefly, and incubated at room temperature for 15 min [49]. Finally, 2 μL of the ligation reaction mixture was used to transform the vector into competent cells.

### Transforming E. coli competent cells with a Cloned pcDNA3.1 (+) vector containing the CAR gene construct

The One Shot TOP10 Chemically Competent E. coli **(Invitrogen, ThermoScientific, USA**) was used for the transformation. Following the manufacturer's instructions, 2 μL of the ligation reaction was added to the competent cells, gently mixed, and incubated on ice for 30 min, followed by 30 s of incubation in a 42 °C water bath without shaking, followed by immediate cooling on ice. Next, 250 μL of pre-warmed Super Optimal broth (S.O.C) medium was added, and the vial was shaken at 37 °C for 1 h at 225 rpm. 100 μL of the transformation mix was spread onto Luria Broth LB agar plates with the appropriate antibiotic using a sterile plastic spreader, and the plates were labeled and incubated overnight at 37 °C. The following day, colonies were selected for further experiments. The transformation process was validated using several controls: a positive control with pcDNA3.1( +) containing a penicillin resistance gene on LB agar with penicillin, a DNA negative control with no pcDNA3.1 plasmid, a cells negative control with no cells, a mock transformation control with un-cloned pcDNA3.1( +), and a competent cells viability control plated on non-selective agar before transformation [50].

### Transduction of activated T cells with a CAR gene construct

Activated CD3/CD28 T cells were transduced with the CAR T construct using Lipofectamine 3000 (***Invitrogen, USA***) [51]. In a 24-well plate, 1 × 10^4 activated CD3/CD28 cells were seeded in 200 µL of Opti-MEM (***Gibco, Life Technologies***) and cultured for 48 h until 90% confluency. Lipofectamine 3000 was diluted by adding 0.3 µL of the reagent to 5 µL of Opti-MEM medium and mixed. A DNA master mix was prepared by diluting 10 µg of DNA in 10 µL Opti-MEM and adding 0.4 µL of P3000 Reagent, followed by vortex mixing. On the second day, diluted DNA (with P3000) and diluted Lipofectamine 3000 were mixed in a 1:1 ratio and incubated for 10–15 min at room temperature. The DNA-lipid complex (10 µL) was added to the cell suspension and incubated at 37 °C and 5% CO2 for 48 h. Transfected cells were visualized and analyzed for transduction efficacy using immunofluorescent staining with a CAR antibody and flow cytometry data analyzed with ***Beckman Coulter Navios EX software ***[52].

### Assessment of cytotoxic effect of CAR T Cells on rituximab resistant (RR-NU-DUL-1) Cells

To evaluate the cytotoxic effect and enhanced Rituximab sensitivity in RR-NU-DUL1 cells, these cells were treated with WEE1-engineered T cells for 72 h, followed by a cell viability assay. The procedure involved seeding 1 × 10^4 RR-NU-DUL1 cells in a 96-well plate for 24 h. Cells were maintained in RPMI-1640 medium (***Gibco, ThermoScientific, USA***) with 10% fetal bovine serum (FBS) and 1% PSA (penicillin G sodium, streptomycin, and amphotericin B). The culture was incubated at 37 °C with 5% CO2. The next day, the medium was replaced, and the cells were treated with 1 × 10^4 activated CD3/CD28 cells, 1 × 10^4 CAR-engineered CD3/CD28 cells, or 1 × 10^4 naive CD3 cells. PBS-treated cells served as the negative control, and cells treated with the IC_50_ of Rituximab (100 µmol/mL) served as the positive control. After 48 h of incubation at 37 °C and 5% CO2, cell proliferation was assessed using the **Vybrant® MTT Cell Proliferation Assay Kit (ThermoSientific, USA**) [53]. MTT solution (20 µL, 1 mg/mL) was added to each well, and the plates were incubated for four hours. The MTT solution was then replaced with 100 µL of SDS-HCL. Cell viability was measured by optical density at 570 nm using a spectrophotometer (***ELx 800; Bio-Tek Instruments Inc., Winooski, VT, USA***).

### Assessment of the apoptotic effect of CAR T cells on Rituximab Resistant (RR- NU-DUL-1) cell line using Annexin/PI staining

One day before the experiment, 1 × 10^6 RR-NU-DUL1 cells were seeded in a 6-well plate and maintained in RPMI-1640 medium (***Gibco, ThermoScientific, USA***) with 10% FBS and 1% PSA (penicillin G sodium, streptomycin, amphotericin B) at 37 °C and 5% CO2. The next day, the medium was replaced, and the cells were treated with 1 × 10^6 CAR T CD3/CD28 cells. The experiment included three groups: negative control (RR-NU-DUL1 cells treated with PBS), RR-NU-DUL1 cells treated with naive CD3 cells, and RR-NU-DUL1 cells treated with CAR-engineered CD3/CD28 cells. After 48 h of incubation, treated cells were isolated using CD3/CD28 Dynabeads, centrifuged, washed with PBS, and resuspended. Apoptosis in RR-NU-DUL1 cells was assessed using the Alexa Fluor® 488 annexin V/Dead Cell Apoptosis Kit (***Invitrogen, ThermoFisher, UK***) [54]. Cells were stained with Alexa Fluor 488 annexin V and PI, incubated, and analyzed by flow cytometry using ***Beckman Coulter Navios EX software***.

### Cell cycle assay

The RR-NU-DUL-1 cells were stained with Vybrant DyeCycle Violet stain (***Invitrogen, USA***). Flow cytometry was used to examine the DNA content distribution during different stages of the cell cycle. One mL of cell suspension in complete media, at a concentration of 1 × 10^6 cells/mL, was mixed with 1 μL of Vybrant® DyeCycle™ Violet stain, resulting in a final stain concentration of 5 μM. The stained cells were incubated at 37 °C for 30 min, protected from light, and kept at 37 °C until acquisition. Data analysis was performed using ***Beckman Coulter Navios EX software*** (version: Navios EX, Beckman Coulter) with ~ 405 nm excitation and ~ 440 nm emission. The population analysis indicated the distribution of apoptotic sub-G1 cells, G0/G1 phase, S phase, and G2/M phase [55].

### Measurement of WEE1 signaling pathway genes expression in activated T cells with a CAR construct using Quantitative Real-time PCR

The effect of transduction with pcDNA3.1-CAR on WEE1 gene suppression in RR-NU-DUL1 cells was investigated by amplifying the WEE1 G2 Checkpoint Kinase genes including "B-cell Leukemia lymphoma gene 2, (BCL2), WEE1, and Cyclin-dependent kinases (CDK1)". Three examined groups are tested, including (1) untreated RR-NU-DUL1 cells, (2) RR-NU-DUL1 cells treated with CD3/CD28( +) cells, and (3) RR-NU-DUL1 cells treated with pcDNA3.1-CAR. Cells were harvested and subjected to RNA extraction using the RNeasy Mini Kit, followed by reverse transcription with the Quantitect RT kit (Qiagen, Hilden, Germany). Amplification of the genes was performed using the QuantiTect primer assay (cat no: 249900) [Hs_WEE1, assay ID: QT00038199; Hs_BCL2, assay ID: QT0025011; Hs-CDK2, assay ID: QT00005586]. The GAPBDH (gene ID: QT00079247) was used as a housekeeper gene for the normalization of gene expression [56], following manufacturer instructions for RNA extraction, reverse transcription, and PCR amplification methods.

### Statistical analysis

The collected data was revised, coded, tabulated, and introduced to a PC using ***GraphPad Prism Software 8.4.2 (San Diego, US)***. The data are presented in Mean, Standard deviation (± SD), and range for parametric numerical data. The independent t-test and the Analysis of variances test (ANOVA), followed by Tukey's tests, were performed to assess the statistical significance of the difference between the means of the two or more two groups, respectively. The P-value ≤ 0.05 was considered significant.

## Results

### CAR T targeting WEE1 inhibits DLBCL cell growth and proliferation

**Fig. 1:**
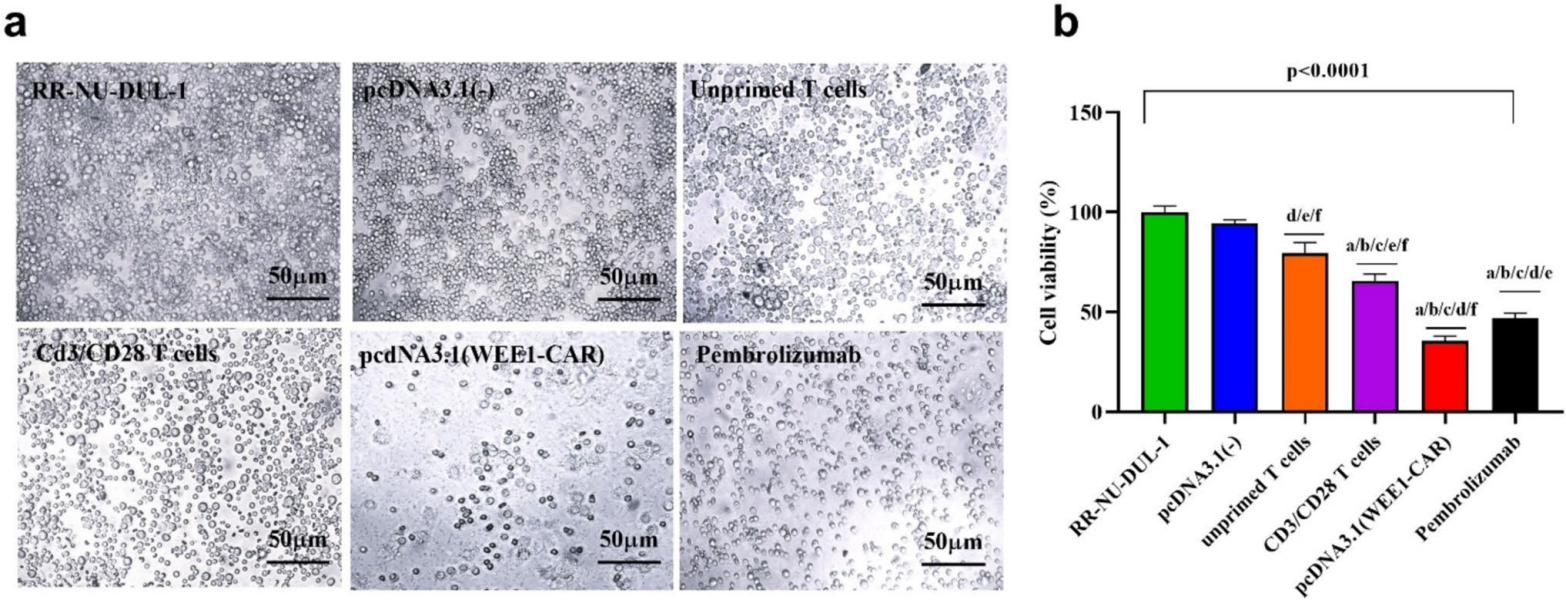
CAR T-cell targeting WEE1 suppresses proliferation in DLBCL cells. (**a**) Untreated RR-NU-DUL-1 cells display 100% viability, Co-Culture of RR-NU-DUL-1 with pcDNA3.1(-) "empty vector", Co-Culture of RR-NU-DUL-1 with unprimed T cells, Co-Culture of RR-NU-DUL-1 with CD3/CD28 activated T cells, Co-Culture of RR-NU-DUL-1 with pcDNA3.1-WEE-CAR, Co-Culture of RR-NU-DUL-1 with 3 μmol/mL of Pembrolizumab. (**b**) Comparative Analysis between the percentage of cell viability at 72 h, determined by MTT assay in RR-NU-DUL-1 cells transfected with pcDNA3.1 vector, pcDNA3.1-CAR, unprimed T cells, CD3/CD28 activated T cells, compared to untreated cells, a: significant difference compared to untreated RR-NU-DUL-1 cells (p < 0.05), b: significant difference compared to cells transfected with empty vector pcDNA3.1 (p < 0.05), c: significant difference compared to cells transfected with unprimed T cells, d: CD3/CD28 activated T cells (p < 0.05), e: cells transfected with pcDNA3.1-WEE1 CAR (p < 0.05), f: cells treated with 3 µmol/mL of Pembrolizumab. ANOVA: Analysis of variances. Data are presented in mean ± SD.RR-NU-DUL-1: Rituximab-resistant NU-DUL-1 cells, CAR: Chimeric antigen receptor

A comparative analysis of RR-NU-DUL-1 cell viability after 72 h of transfection with WEE1-CAR, Pembrolizumab, CD3/CD28-activated T cells, and unprimed T cells was performed using an MTT assay. WEE1-CAR transfected cells exhibited the lowest viability (35.59%), followed by Pembrolizumab (47.49%) and CD3/CD28-activated T cells (68.96%), while unprimed T cells showed 84.36% viability. ANOVA and Tukey's test revealed significant differences between the groups (F = 188.3, p = 0.0001), indicating that WEE1-CAR cells induced the highest cytotoxic effect compared to untreated cells (mean difference: 64.40, p < 0.0001). Results are detailed in Tables 1 and Fig. 1.

**Table 1:** Mean of cell viability (%) in different groups of RR-NU-DUL-1 cells at 72 h after transfection

Group	Mean ± SD	range	Statistics
RR-NU-DUL-1	100 ± 3.06	98.2 – 104	F = 188.3, *p* = 0.0001 [HS]
pcDNA3.1(-)	94.5 ± 1.59	92.7 – 95.6	
Unprimed T cells	79.5 ± 5.22	74.0 – 84.4	
CD3/CD28 T cells	65.4 ± 3.55	61.9 – 69.0	
pcDNA3.1(WEE1-CAR)	35.6 ± 2.39	32.8 – 37.0	
Pembrolizumab	46.9 ± 2.47	44.2 – 49.1	

*pcDNA3.1(-)*: empty vector, *CAR*: chimeric antigen receptor, *F*: ANOVA test value, *HS*: high significant differences (*p* < 0.01)

### CAR T targeting WEE1 promotes apoptosis of DLBCL cells

**Fig. 2:**
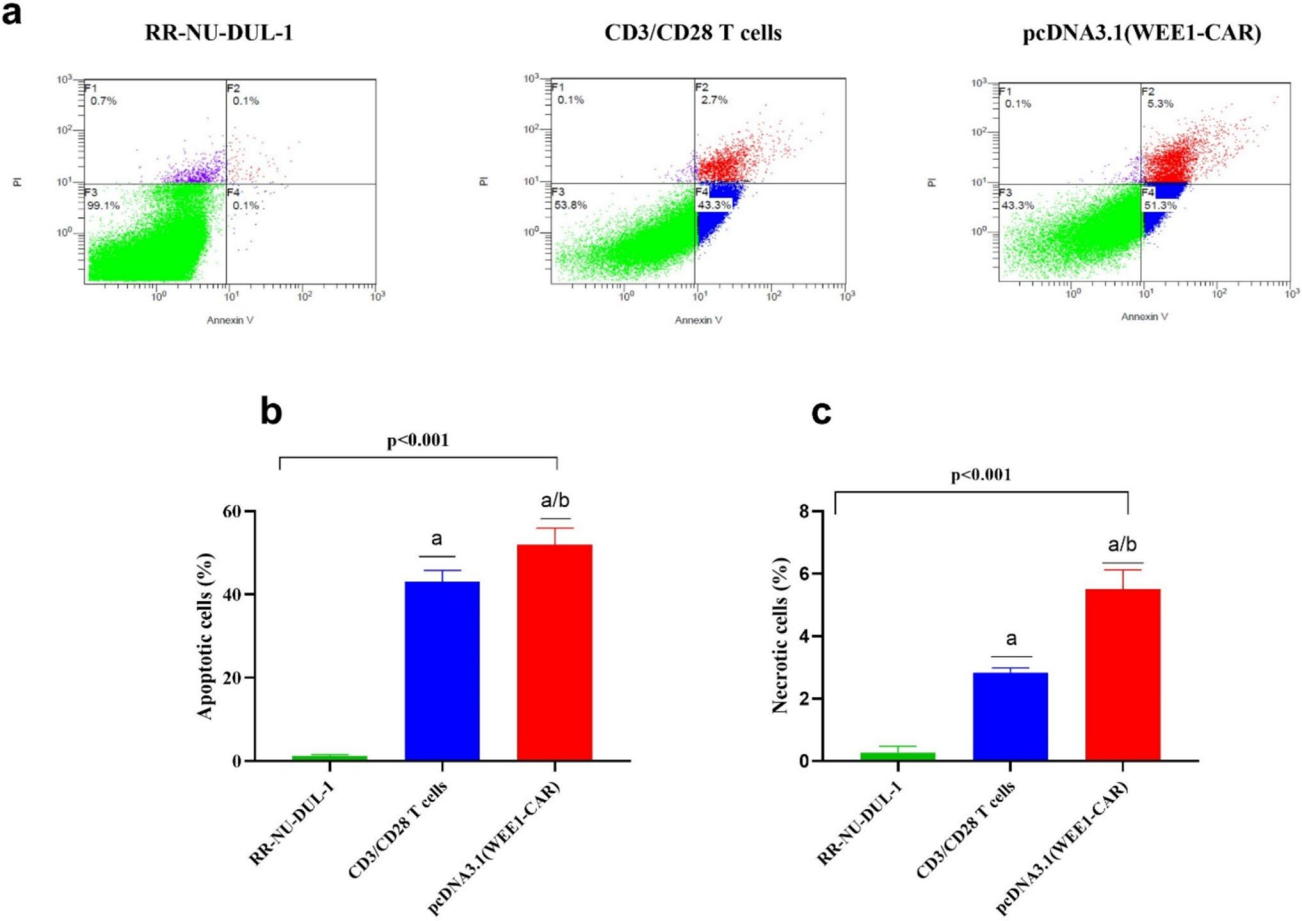
Early and late apoptosis in rituximab-resistant cell lines after treatment for 72 h. Cells are stained with annexin V/PI staining and analyzed using flow cytometry. (**a**) presents the four-quadrant histogram for RR-NU-DUL-1 cells "untreated", co-cultured with CD3/CD28 activated T cells, and cells cocultured with pcDNA3.1 (WEE1-CAR), using a Navios EX Flow Cytometer (Beckman Coulter, USA). (**b**) ANOVA comparative test for the percentage of apoptotic cells (early and late apoptosis), (**c**) ANOVA comparative test for the percentage of necrotic cells. a: significant difference compared to untreated RR-NU-DUL-1 cells (*p* < 0.01), b: significant difference compared to cells transfected with CD3/CD28 activated T cells (*p* < 0.01), ANOVA: Analysis of variances. Data are presented in mean ± SD.RR-NU-DUL-1: Rituximab-resistant NU-DUL-1 cells, CAR: Chimeric antigen receptor, FITC, fluorescein 5(6)-isothiocyanate; PI, propidium iodide

The % of apoptotic and necrotic cells was measured in RR-NU-DUL-1 cells after 72-h treatments with pcDNA3.1 (WEE1-CAR) and activated CD3/CD28 T cells, compared to untreated controls. The results indicated a higher percentage of apoptotic cells in pcDNA3.1 (WEE1-CAR) treated cells (5%) than in cells treated with activated CD3/CD28 T cells (0.5%) and untreated cells. Necrosis was more prominent in pcDNA3.1 (WEE1-CAR) treated cells (11.7%) compared to those treated with activated CD3/CD28 T cells (4.9%). These findings are illustrated in Fig. 2.

ANOVA analysis revealed a significant difference in apoptosis rates among the three groups. Treatment with pcDNA3.1 (WEE1-CAR) was significantly associated with a higher percentage of apoptotic cells compared to both CD3/CD28 activated T cells and untreated cells (*p* < 0.0001). Additionally, treatment with CD3/CD28 activated T cells also showed a significantly higher apoptosis rate compared to untreated cells (*p* < 0.0001).

Necrosis rates were also compared using ANOVA followed by Tukey's multiple comparisons test, revealing significant differences between the groups (F = 121.4, *p* = 0.0001). The highest necrosis percentage was observed in RR-NU-DUL-1 cells treated with pcDNA3.1 (WEE1-CAR), followed by those treated with activated CD3/CD28 T cells. A significant difference in necrosis was detected between cells treated with pcDNA3.1 (WEE1-CAR) and those treated with activated CD3/CD28 T cells (*p* < 0.001). Furthermore, both treatment groups showed significantly higher necrosis rates compared to untreated cells. Detailed results are presented in Fig. 2.

### CAR T targeting WEE1 induces a cell cycle arrest in Rituximab-resistant DLBCL cells

**Fig. 3:**
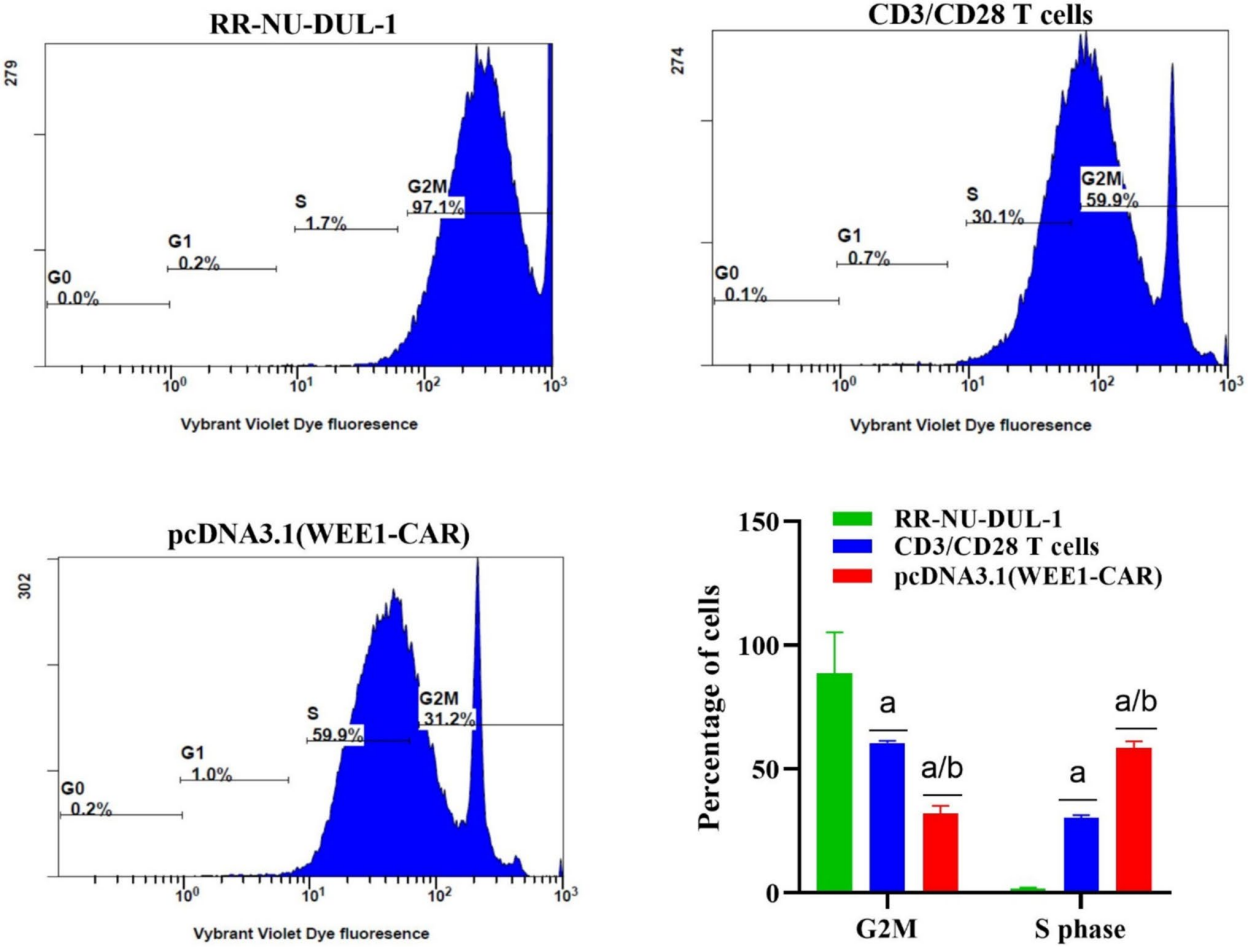
Distribution of cell cycle phases in the RR-NU-DUL-1 cells, cells treated with CD3/CD28 T cells, and cells treated with pcDNA3.1 (WEE1-CAR) for 72 h

We examined the cell cycle distribution across the three groups tested. The treatment with pcDNA3.1 (WEE1-CAR) led to an increase in the proportion of cells in the G2/M phase (31.2%) and S phase (59.9%), suggesting either premature entry into mitosis or extended mitotic arrest. This was in comparison to RR-NU-DUL-1 cells treated with CD3/CD28 T cells (p = 0.001) and untreated RR-NU-DUL-1 cells **(**Fig. 3).

### CAR T targeting WEE1 decreases the expression of the WEE1 gene in Rituximab-resistant DLBCL cells

**Fig. 4:**
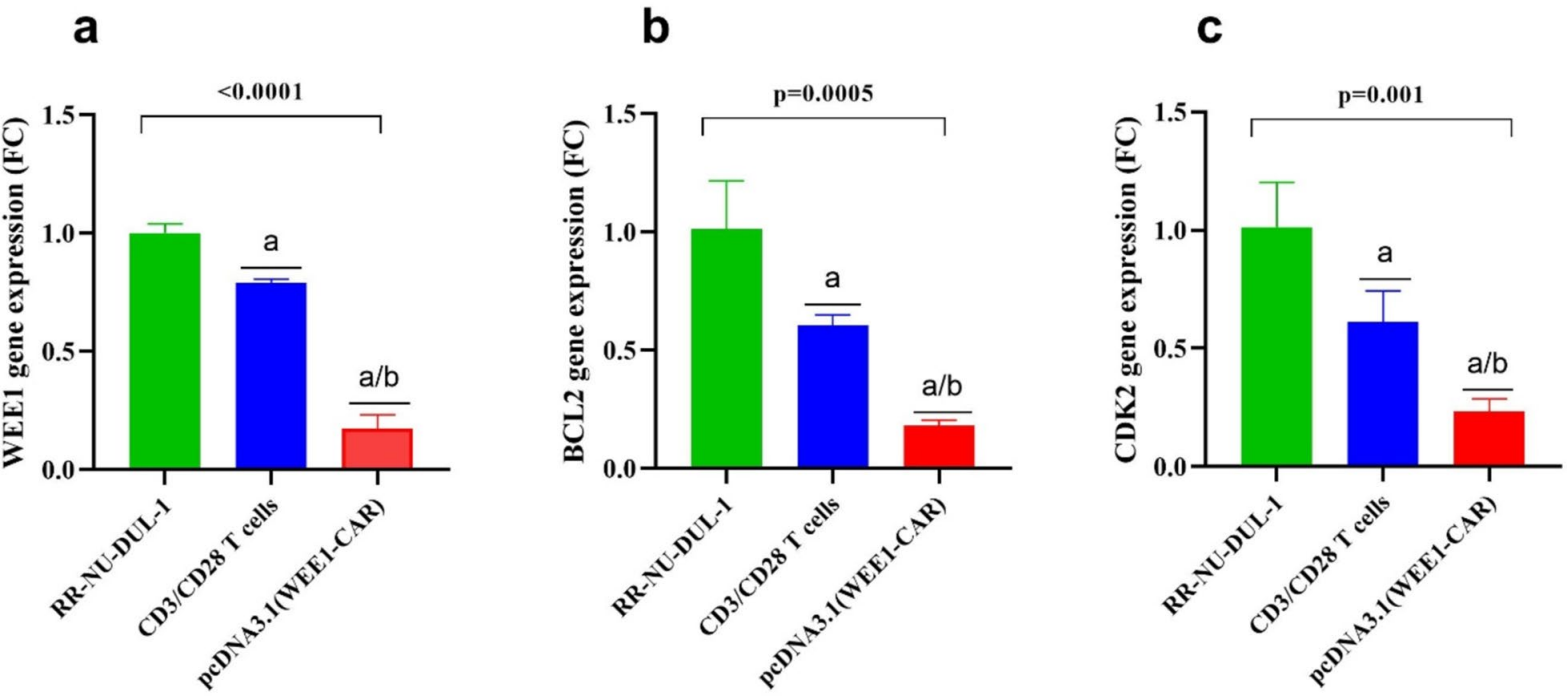
Gene expression in rituximab-resistant cell lines treated for 72 h. (**a**) presents the WEE1 gene expression, (**b**) BCL2 gene expression, (**c**) CDK2 gene expression. The three genes are measured in three groups "RR-NU-DUL-1 cells "untreated", co-cultured with CD3/CD28 activated T cells, and cells cocultured with pcDNA3.1 (WEE1-CAR). a: significant difference compared to untreated RR-NU-DUL-1 cells (*p* < 0.05), b: significant difference compared to cells treated with CD3/CD28 activated T cells (*p* < 0.05). Data are presented in mean ± SD. ANOVA: Analysis of variances, RR-NU-DUL-1: Rituximab-resistant NU-DUL-1 cells, CAR: Chimeric antigen receptor

To assess the cytotoxic impact of WEE1-CAR on RR-NU-DUL-1 at the genetic level, three genes "B-cell Leukemia lymphoma gene 2, (BCL2), WEE1, and Cyclin-dependent kinases (CDK1) were analyzed in RR-NU-DUL-1 cells treated with pcDNA3.1 (WEE1-CAR) or CD3/CD28 for 72 h using Syber green-based qPCR. WEE1 gene expression was measured and normalized to that in untreated cells, with results shown as fold change (Table 2). The findings indicated that WEE1 gene expression showed a significant reduction in RR-NU-DUL-1 cells after treatment with pcDNA3.1 (WEE1-CAR), compared to untreated cells as well as cells treated with CD3/CD28 activated T cells Table 2, Fig. 3a). Similar results were obtained for the expression of BCL2 (Table 2, Fig. 4b) and CDK2 (Table 2, Fig. 4c) genes, WEE1-CAR T cells treatment was associated with significant reduction in RR-NU-DUL-1 cells (P < 0.01), compared to those treated with CD3/CD28 T cells and untreated cells (Fig. 4).

**Table 2:** Mean values of gene expression (FC) in different groups of RR-NU-DUL-1 cells at 72 h after transfection (ANOVA)

Group	WEE1 (FC)	BCL2 (FC)	CDK2 (FC)
RR-NU-DUL-1	1.0 ± 0.038	1.0 ± 0.204	1.0 ± 0.189
CD3/CD28 T cells	0.79 ± 0.014	0.605 ± 0.044	0.612 ± 0.132
pcDNA3.1(WEE1-CAR)	0.173 ± 0.05	0.180 ± 0.02	0.234 ± 0.05
Statistics	F: 320, *p* < 0.0001	F: 35.37, *p* = 0.0005	F: 24.44, *p* = 0.0013

*CAR* chimeric antigen receptor, *F* ANOVA test value, *CDK2* Cyclin-dependent kinases 2, *BCL2* B-cell Leukemia lymphoma gene 2, *FC* fold change in gene expression

## Discussion

Rituximab is the primary treatment for DLBCL [57], but resistance often arises [1, 58, 59]. Understanding the molecular mechanisms behind this resistance is crucial for designing effective new treatments [1]. WEE1, overexpressed in DLBCL, regulates the cell cycle by inhibiting the G2/M transition and has shown potential as a target [60, 61]. The development of cell-based therapies emphasizes the potential of engineered T cells to target specific cell clones, offering a promising strategy to combat drug resistance [62].

In the context of DLBCL treated with R/CHOP, our focus on rituximab-resistant cells is due to the fact that rituximab resistance is specifically associated with immunotherapy failure through CD20 and BCL2 signalling pathways, whereas resistance to the R/CHOP regimen involves more complex, multifactorial mechanisms [63]. CAR T-cell therapy, while promising, is also limited by its reliance on consistent antigen expression. Loss or downregulation of the target antigen can reduce CAR T-cell efficacy, presenting challenges in managing resistant DLBCL [64].

This study assesses the effectiveness of WEE1-specific Chimeric Antigen Receptor (CAR) T cells against refractory Diffuse Large B-cell Lymphoma (DLBCL) using in vitro experiments on Rituximab-resistant DLBCL cell line (RR-NU-DUL-1). Fifth generation CAR T cells targeting WEE1 were computationally constructed. T cells isolated from PBMNCs were expanded, transduced with the WEE1-CAR gene, and co-cultured with RR-NU-DUL-1 cells for 72 h. Their efficacy was compared to untreated cells and cells co-cultured with activated CD3/CD28 T cells using cell proliferation, apoptosis, cell cycle, and gene expression assays for WEE1, BCL2, and CDK2 genes.

The need for fifth-generation CARs arises from the limitations of current CAR T-cell therapies, where over 60% of patients relapse post-treatment. Fifth-generation CARs incorporate additional signalling domains to enhance T-cell persistence, proliferation, and functionality, addressing the challenge of relapse [65]. These improvements aim to provide more durable responses to overcome the post-CARs relapse, particularly in relapsed/refractory DLBCL, by ensuring sustained anti-tumor activity, thus potentially improving long-term outcomes in these high-risk patients [66].

The study demonstrates the promising potential of WEE1-CAR T cells in treating DLBCL, especially in cells resistant to Rituximab. The research shows that WEE1-CAR T cells significantly inhibit the growth and proliferation of the RR-NU-DUL-1 DLBCL cell line. MTT assays revealed that WEE1-CAR transfected cells have the lowest viability (35.59%) compared to cells treated with Pembrolizumab (47.49%), CD3/CD28-activated T cells (68.96%), and unprimed T cells (84.36%). These results highlight the potent cytotoxic effect of targeting the WEE1 gene in reducing DLBCL cell viability. Previous studies by Lucy A et al. (2019) [67] reported selective antitumor activity of WEE1 inhibitors in non-germinal center B-cell DLBCL cell lines, demonstrating that WEE1 inhibitors induce cytotoxicity through aberrant CDK1 and CDK2 activation [68], disrupting the DNA-damage G2–M checkpoint [69]. Further studies linking these biomarkers with high WEE1 inhibitor sensitivity could extend the use of these targeted agents to other tumor types [28, 70].

The study also shows that WEE1-CAR T cells induce a higher apoptosis rate in DLBCL cells. Flow cytometry analysis indicates that 5% of WEE1-CAR T cell-treated cells undergo apoptosis, compared to 0.5% for CD3/CD28-activated T cells and untreated cells. Necrosis is also higher in WEE1-CAR treated cells (11.7%) versus activated CD3/CD28 T cells (4.9%). These findings suggest that WEE1-CAR T cells inhibit cell growth and promote cell death via apoptotic and necrotic pathways. Additionally, WEE1-CAR T cells cause cell cycle arrest, with significant increases in the G2/M phase (31.2%) and S phase (59.9%), indicating disrupted cell cycle progression, leading to extended mitotic arrest or premature mitosis, thereby inhibiting cancer cell proliferation and enhancing CAR T cell efficacy.

WEE1 triggers DNA damage and forces premature entry into mitosis in DLBCL, increasing the cells' dependence on BCL-2 [29]. The resulting G2/M cell cycle arrest and DNA damage stress heighten DLBCL cells' reliance on anti-apoptotic proteins. Therefore, combining genotoxic or cell cycle-disrupting agents with anti-apoptotic inhibitors could be particularly effective for treating genomically unstable cancers like DLBCL, meriting further clinical investigation [60]. Early clinical trials using WEE1 inhibitors in other cancers show promising results, highlighting the potential of this strategy [71].

Since WEE1 regulates the CDK1-cyclinB complex, its inhibition leads to premature mitochondrial fragmentation and destabilizes the connection between cell division and mitochondrial function [25, 72]. Additionally, DNA damage activates ATM, which phosphorylates BID, triggering apoptosis [73]. This reveals a new link between the cell cycle, DNA damage, and mitochondrial apoptosis [73]. Although WEE1 inhibition has not been extensively tested in other cancers, its fundamental role suggests similar responses could occur. Combining WEE1 inhibitors with BH3 mimetics holds promise for dose reduction therapy in treatment-naïve patients or as a sensitizer in tumors resistant to WEE1 inhibitors [29].

At the molecular level, WEE1-CAR T cells significantly reduce the expression of the WEE1 gene in RR-NU-DUL-1 cells. Quantitative PCR analysis reveals a substantial decrease in WEE1 gene expression, as well as reductions in BCL2 and CDK1 gene expressions, after treatment with WEE1-CAR T cells compared to untreated cells and those treated with CD3/CD28 T cells. This downregulation of key genes essential for cell survival and proliferation underscores the therapeutic potential of WEE1-CAR T cells in targeting refractory DLBCL [74].

Recent studies have investigated the cytotoxic effects of WEE1 inhibitors either alone or in conjunction with checkpoint proteins or chemotherapy. Others have focused on evaluating the safety, efficacy, and stability of CAR T cells. However, there has been no exploration into combining these two approaches, which underscores the novelty of this study.

WEE1-CAR T cells may offer advantages over CD19-directed therapies by targeting a distinct mechanism—cell cycle regulation and DNA repair pathways—rather than relying solely on CD19 expression. This could be beneficial in cases where tumours develop resistance through CD19 loss or downregulation, a known cause of relapse in current CAR T-cell therapies [75]. WEE1-CAR T cells could potentially overcome this challenge, providing a complementary approach and reducing relapse rates in relapsed/refractory DLBCL.

In conclusion, the study was the first to introduce a WEE1 targeting CAR T cells that shows WEE1-specific CAR T cells are promising for treating refractory DLBCL by inhibiting cell growth, inducing apoptosis, and disrupting cell cycle progression. Further research and clinical trials are crucial to fully realize their potential and improve patient outcomes. These findings highlight WEE1 as a novel therapeutic target for CAR T cell-based therapy, offering personalized treatment strategies for DLBCL patients.

The results of this study are crucial for advancing new treatments for refractory DLBCL. WEE1-CAR T cells employ a multifaceted strategy in cancer therapy by inhibiting cell proliferation, triggering apoptosis, inducing cell cycle arrest, and suppressing key survival genes. Together, these mechanisms contribute significantly to the powerful anti-cancer effects observed in the research.

However, translating these findings into clinical practice poses several challenges. Firstly, the safety and effectiveness of WEE1-CAR T cells require comprehensive evaluation in both preclinical and clinical trials [76]. Monitoring potential off-target effects and assessing the overall toxicity profile are essential to ensure patient safety [77]. Moreover, optimizing the delivery and persistence of CAR T cells in the body is critical for achieving sustained therapeutic benefits [78].

The study's limitations include its reliance on in vitro experiments with DLBCL cell lines, which may not fully reflect in vivo conditions due to the complex tumor microenvironment and interactions within the human body. Specifically, focusing on the RR-NU-DUL-1 cell line limits the generalizability of findings across different DLBCL subtypes and other B-cell malignancies. Additionally, the study lacks detailed insights into the long-term safety and toxicity profiles of WEE1-CAR T cells.

Future research should prioritize conducting in vivo studies using animal models to evaluate the efficacy and safety of WEE1-CAR T cells. These studies will provide crucial information on pharmacokinetics, biodistribution, and potential toxicities. Following successful preclinical evaluations, early-phase clinical trials should be conducted to assess the therapeutic potential of WEE1-CAR T cells in refractory DLBCL patients. Enhancing CAR T cell design to improve specificity, persistence, and efficacy, as well as combining WEE1-CAR T cells with other therapeutic approaches like checkpoint inhibitors or modifying the tumor microenvironment, represents promising avenues for future investigation.

## Supplementary Information

Below is the link to the electronic supplementary material.

Supplementary file1 (XLSX 11 KB)

## Data Availability

No datasets were generated or analysed during the current study.
